# Multilayers of Renewable Nanostructured Materials with High Oxygen and Water Vapor Barriers for Food Packaging

**DOI:** 10.1021/acsami.2c07579

**Published:** 2022-06-21

**Authors:** Eva Pasquier, Bruno D. Mattos, Hanna Koivula, Alexey Khakalo, Mohamed Naceur Belgacem, Orlando J. Rojas, Julien Bras

**Affiliations:** † Université Grenoble Alpes, CNRS, Grenoble INP (Institute of Engineering), LGP2, F-38000 Grenoble, France; ‡ Department of Bioproducts and Biosystems, School of Chemical Engineering, Aalto University, P.O. Box 16300, Aalto, FIN-00076 Espoo, Finland; § Department of Food and Nutrition and Helsinki Institute of Sustainability Science, University of Helsinki, Agnes Sjöobergin katu 2, P.O. Box 66, FIN-00014 Helsinki, Finland; ∥ VTT Technical Research Centre of Finland Ltd., Tietotie 4E, P.O. Box 1000, FIN-02044 Espoo, Finland; ⊥ Institut Universitaire de France (IUF), F-75000 Paris, France; # Bioproducts Institute, Department of Chemical and Biological Engineering, Departments of Chemistry and Departments of Wood Science, University of British Columbia, 2360 East Mall, Vancouver, British Columbia V6T 1Z3, Canada

**Keywords:** cellulose nanofibers, wax, lignin particles, layered biopolymers, sustainable films, biobased packaging

## Abstract

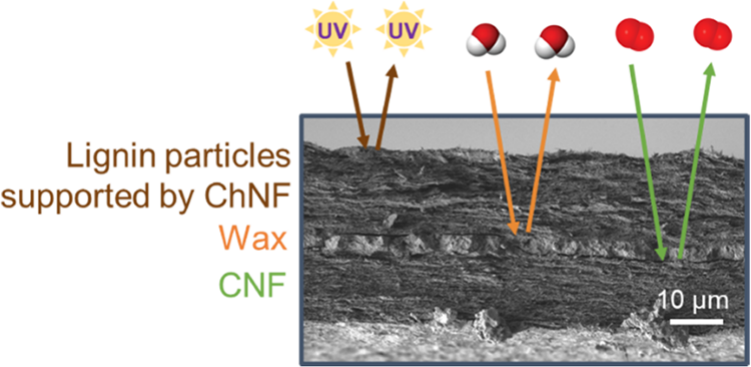

Natural biopolymers have become key players in the preparation of biodegradable food packaging. However, biopolymers are typically highly hydrophilic, which imposes limitations in terms of barrier properties that are associated with water interactions. Here, we enhance the barrier properties of biobased packaging using multilayer designs, in which each layer displays a complementary barrier function. Oxygen, water vapor, and UV barriers were achieved using a stepwise assembly of cellulose nanofibers, biobased wax, and lignin particles supported by chitin nanofibers. We first engineered several designs containing CNFs and carnauba wax. Among them, we obtained low water vapor permeabilities in an assembly containing three layers, i.e., CNF/wax/CNF, in which wax was present as a continuous layer. We then incorporated a layer of lignin nanoparticles nucleated on chitin nanofibrils (LPChNF) to introduce a complete barrier against UV light, while maintaining film translucency. Our multilayer design which comprised CNF/wax/LPChNF enabled high oxygen (OTR of 3 ± 1 cm^3^/m^2^·day) and water vapor (WVTR of 6 ± 1 g/m^2^·day) barriers at 50% relative humidity. It was also effective against oil penetration. Oxygen permeability was controlled by the presence of tight networks of cellulose and chitin nanofibers, while water vapor diffusion through the assembly was regulated by the continuous wax layer. Lastly, we showcased our fully renewable packaging material for preservation of the texture of a commercial cracker (dry food). Our material showed functionality similar to that of the original packaging, which was composed of synthetic polymers.

## Introduction

Replacement policies for petroleum-based plastics and the current regulations on single-use plastics are incentivizing the development of biobased materials for packaging.^1^ Biobased plastics include a wide range of materials, such as polymers obtained from biobased monomers and biosynthesized polymers (biopolymers) that can be extracted from natural and renewable resources.^2^ Biopolymers extracted from nature offer a better end of life due to their biodegradability, which is not necessarily the case of other biobased plastics such as bio-polyethylene, bio-polypropylene, or bio-PET. Biopolymer-based packaging can degrade in natural environments within a short time when not chemically modified.^3^ Cellulose fibers can be extracted from plant biomass, which offers advantages in terms of geographic availability.^4^ However, up to now, natural fiber-based materials have had limited applications in food packaging because of their high gas permeability.^5^

Cellulose nanofibers (CNF) are cellulose colloids that can lead to materials with high oxygen and grease barrier properties.^6,7^ They are obtained by mechanical fibrillation of cellulose fibers and lead to nanofibers that are a few micrometers long and only tens of nanometers wide.^8^ CNF can be assembled into self-standing films or coated on different substrates such as paper boards or plastics (PLA, PET).^9−12^ Upon drying, the entanglement of the high-aspect ratio nanofibers leads to high internal cohesion within the CNF network. This cohesive film forms a packed structure that has high air, oxygen, and grease barrier.^7,9^ However, cellulose is hydrophilic and very sensitive to moisture, which exponentially increases the oxygen transmission rate (OTR) of CNF films at 50–80% relative humidity because of the swelling effect of water molecules on the nanofibrillar network.^13,14^ Moreover, the typical water vapor transmission rate (WVTR) of CNF ranges between 100 and 500 g/m^2^·day at 50% RH, which is too high for most commercial applications.^15^

Many parameters can influence water vapor permeability (WVP) of cellulose films including density, pore structure, surface chemistry, crystallinity, and the parameters chosen for film drying.^2,15^ Sharma et al. have shown that heat treatment (3 h at 175 °C) of CNF films decreased WVP by a factor of 2,^16^ which was explained by a decrease in the porosity of the film that slowed down gas diffusion and by an increase in cellulose crystallinity that limited water vapor penetration in the CNF. However, heat treatments induced brittleness in CNF films. WVTR of CNF films has been also reduced by applying hydrophobic coatings. The CNF film when dipped in melted paraffin displayed a decrease of WVTR from 600 to 40 g/m^2^·day as wax formed a continuous hydrophobic layer on both sides of the film.^17^ Multilayer systems were also proposed by coating paper with CNF and shellac wax to decrease the gas permeability of paper.^18^ WVTR was decreased from 50–70 to 6–8 g/m^2^·day with the presence of 10 μm of shellac coating. Among the different waxes, carnauba wax is a plant-based wax with low water vapor permeability, and despite its lipidic nature, it also contains hydrophilic groups such as esters and hydroxyls, which could improve interactions with CNFs.^19^

**Figure 1:**
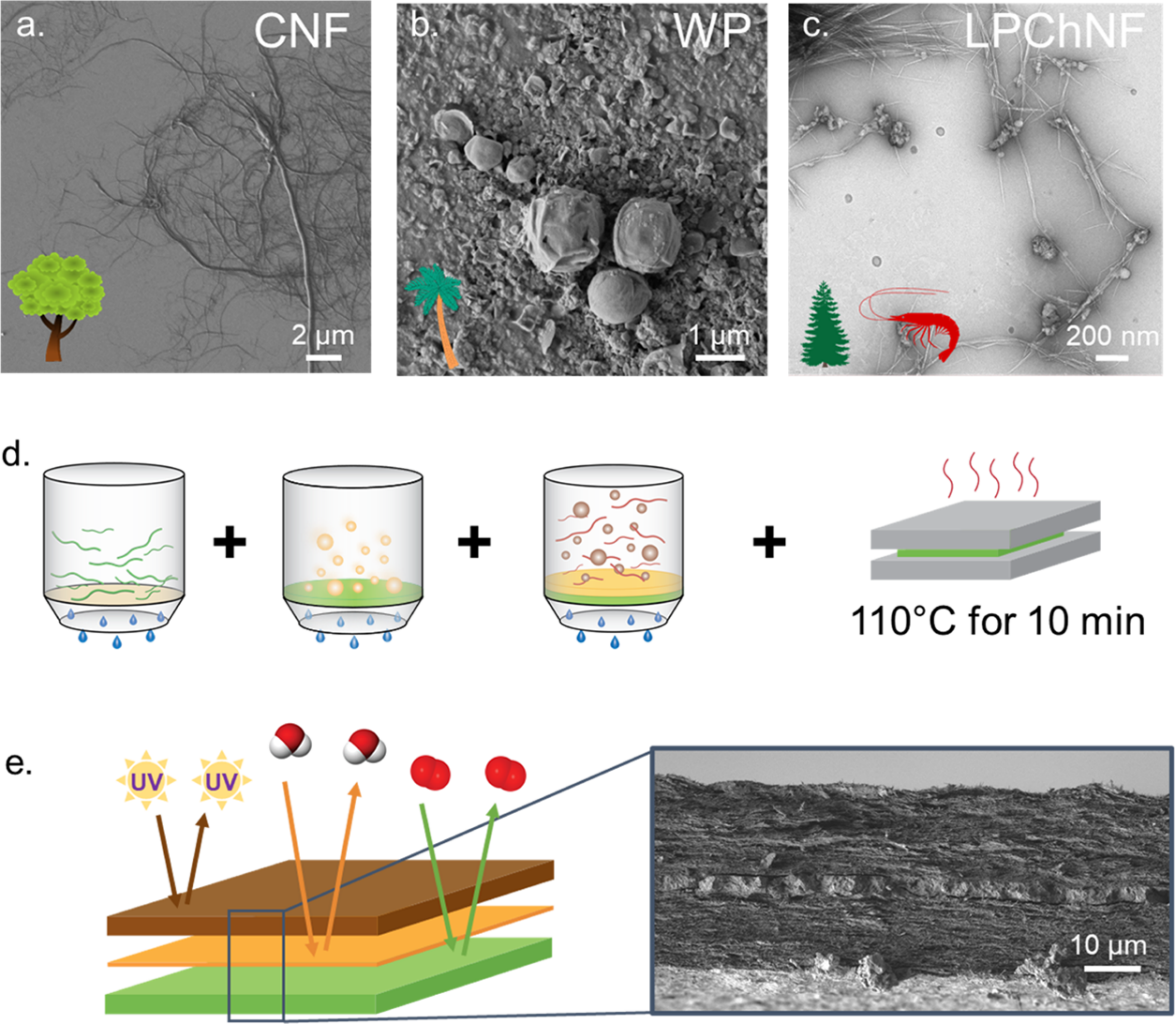
SEM image of different colloids used in the multilayer film: (a) cellulose nanofibers (CNF), (b) wax particles (WP), and (c) lignin particles with chitin nanofibers (LPChNF). Filtration and drying steps used to produce the multilayer film (d) and scheme of the produced multilayer with UV light, water vapor, and oxygen barrier properties associated with an SEM image of the cross section (e).

Inspired by currently commercialized multilayered packaging materials, a completely renewable multilayered film displaying high oxygen and water vapor barrier properties was fabricated by engineering layers of carnauba wax and cellulose nanofibers to display specific and complementary barrier functions. To additionally obtain a barrier against UV light and antioxidant properties, we incorporated a third layer composed of lignin nanoparticles immobilized in a chitin nanofiber matrix.^20,21^ Therefore, we produced a multilayer film with three layers designed especially to impose barriers against oxygen, water vapor, and UV light. Multilayered materials are widely used in the food packaging industry because no single material can provide all the requirements for protecting foods, varying, for example, from mechanical properties to high barriers, printability, and sealability. The major drawback of the current multilayer materials is their end of life, as each additional layer impacts the recyclability or biodegradability of the whole packaging.^22^ Herein, we use unmodified biobased materials that when combined together display multiple barrier features while maintaining their biodegradability. Multilayers of CNF, wax, and chitin nanofibers containing lignin nanoparticles (LPChNF) were prepared (Figure 1). Each layer has a specific barrier: oxygen by CNF, water vapor by wax, and UV light by LPChNF. The precursors were combined in the wet state with layer-by-layer filtration and dried as one film (Figure 1d). In this work, the configuration of the wax layer was studied thoroughly using different processes to incorporate wax into a CNF film aiming at low water vapor permeability. Then, multilayer films were prepared, and their morphology was assessed along with their oxygen, water vapor, grease, and UV barriers. Herein, we achieved a high gas barrier in a single structure, which was enabled by stepwise layering of unmodified biobased materials.

## Experimental Section

### Materials

Technical kraft lignin (Indulin AT) from softwood was purchased from MeadWestvaco. Cellulose nanofibers (CNF) were prepared by microfluidization (M-110P, Microfluidics, Inc.) of never-dried bleached kraft hardwood pulp with six passes at 2000 bar with 200–100 μm chambers. Chitin nanofibers (ChNF) were prepared from dried shrimp chitin flakes purchased from Sigma-Aldrich. First, purification of the chitin was carried out following the method reported by Pasquier et al.^23^ Briefly, demineralization (0.25 M HCl, 2 h, room temperature), deproteinization (1 M NaOH, 4 h, 50 °C), and chlorine bleaching (80 °C, 2 h) were done with washing steps in between. The fiber suspension was then acidified to obtain a final acetic acid concentration of 0.1%. Then, the fibers were mechanically fibrillated using a high-shear homogenizer as a pretreatment for 10 min followed by microfluidization (M-110P, Microfluidics, Inc.) with one pass at 1500 bar with 400 and 200 μm chambers and six passes at 2000 bar with 200 and 100 μm chambers.

Lignin particles were prepared *in situ* with ChNF following the procedure described elsewhere.^21^ For this purpose, a solution of lignin was prepared at 2 g/L in acetone/water (9:1). The solution was then filtered on paper filter, and the purified solution was dropped in a suspension of ChNF at 0.5%. The final lignin content was 9% of the total dry mass (ChNF + lignin). The suspension was dialyzed against distilled water to remove residual acetone.

Carnauba wax, acetic acid, calcium chloride (CaCl_2_), HCl, NaOH, NaClO_2_, and “Oil Red O” (1-[2,5-dimethyl-4-(2,5-dimethylphenylazo)phenylazo]-2-naphthol or Solvent Red 27) were purchased from Sigma-Aldrich. The crackers were acquired from the local supermarket.

### Wax Particle Suspension Preparation

Wax particles were prepared following Lozhechnikova et al.^24^ Briefly, wax was melted at 100 °C in distilled water at 1 g/L. When the wax had melted completely, the solution was sonicated at 50% amplitude (45 W) for 5 min, and the emulsion was then cooled rapidly in an ice bath under stirring. The suspension was filtrated on a 90 μm nylon mesh before use.

Nonspherical particles with a diameter of 367 ± 21 nm and ζ-potential of −43 ± 2 mV were obtained (Figure S1b,c). The particle yield was ca. 75%, with bigger wax residues being retained in the filter after the cooling stage. The WP suspension was stable over 6 months, with the average particle size being constant within the standard error, and no extensive particle aggregation was noted in the dynamic light scattering (DLS) measurement (Figure S1c).

### Characterization of the Wax Nanoparticle Suspensions

#### Morphology of Nanoparticles

The wax suspension was dropped on silica and dried overnight. A Au/Pd coating of 4 nm was sputtered on the sample before observation with a Zeiss Sigma VP scanning electron microscope (SEM) operating at 2 kV. Several images were taken, and the most representative one is shown.

#### Hydrodynamic Size Measurement

A dynamic light scattering device (Malvern Zetasizer Nano) was used to measure the wax particle size. Time stability was assessed by measuring the particle size at different times: the day of preparation, 1 week, 2 weeks, 1 month, 2 months, 3 months, and 6 months after the preparation. The measurement was done in duplicate.

#### Surface Charge Measurement

The ζ-potential of the WP was measured with a Malvern Zetasizer Nano using a dip cell. The suspension was diluted to 0.05% and conductivity was adjusted to 0.13 mS/cm with diluted NaCl. The measurement was done in triplicate.

### Preparation of the CNF Film Containing Wax

The CNF suspension was diluted to 0.2% and homogenized for 2 min with a high-shear homogenizer (Ultra-Turax) at room temperature (RT) prior to filtration. The CNF content was kept constant between the different films to obtain a film with a grammage of 40 g/m^2^ and a wax content of 9 wt % of the total mass for all the films; see Table S1 for exact masses. Films were prepared with a vacuum filtration unit, and poly(vinylidene fluoride) (PVDF) membranes with a pore size of 0.45 μm were used as filters. All suspensions were bath-sonicated for 2 min prior to filtration. Figure S2 shows the six different films produced in this work. The reference film contains only CNF and will be called “CNF”. After filtration, a second PVDF membrane was placed on top of the film before drying. All the films were dried in two steps; the first one consisted of pressing at 100 bar and heating at 100 °C for 10 min followed by 35 min cooling to RT under similar pressure. The films were then left to dry overnight at RT under a 5 kg weight.

The second film was a mix of CNF and WP. Both suspensions were mixed before filtration and filtered together; it will be later referred as “Mix”.

The third film, called “top layer”, was produced by filtering first the CNF suspension, and when no visible water remained, the WP suspension was poured over and filtered. For the drying of the top layer film, a Teflon plate was placed on top of the film to avoid leakage of the wax while melting.

The fourth film was composed of a WP layer positioned in between two CNF layers, following the same method as used in the top layer assembly, but the film was produced by filtration of first half of the CNF, then the WP suspension, and finally the second half of the CNF. This film will be referred as “Sandwich”.

Another Sandwich film with a small amount of CNF-WP mix (9% CNF relative to WP weight) was prepared; it is called “Sandwich WP_CNF_”.

Control of the temperature and the time of heating during pressing are key parameters for the formation of a continuous layer of wax. Preliminary experiments showed that lower temperatures and/or shorter times were not enough for the wax to melt and to form a dense layer, while higher temperatures and/or longer times completely melted the wax and formed noncontinuous, coalesced patches of wax across the film. The presence of water during drying and CNF in the WP layer could also influence the melting of the wax.

### Multilayer Film Preparation

The multilayer film was prepared using the same method as the Sandwich film but by replacing the top CNF suspension by the LPChNF suspension to add the UV barrier feature. The amount of nanofibers was kept constant within the films (Table S1). The chitin nanofibers containing the lignin particle suspension (LPChNF) were diluted to 0.2% with diluted acetic acid. Before filtration, the suspension was bath-sonicated for 5 min to remove bubbles.

A film containing only LPChNF was also prepared as a reference for testing the mechanical properties.

### Characterization of the Films

All films were conditioned at 50% relative humidity (RH) and 23 °C for at least 24 h before any characterization.

#### Apparent Density

Films were weighed at 23 °C and 50% relative humidity, and their thickness was measured with a thickness tester for paper and board (Lorentzen and Wettre). Due to the pressing during the drying process, the films had a smooth surface; hence, the thickness could be measured with a thickness tester or paper. Films were circular, and their diameter was measured with a regular ruler. The density was calculated according to the following equation
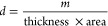
1where *m* represents the mass of the film in g and thickness and area are measured in μm and cm^2^, respectively.

#### Mechanical Properties

Tensile tests were performed with an Instron 5944 with a 100 N load cell at a strain rate of 1 mm/min. The initial gap between the clamps was set at 30 mm, and the samples were cut as strips with dimensions of 50 × 5 mm^2^. The width of the samples was precisely measured with a caliper before each test. The strips were glued in a paper frame to avoid fracture at the clamp edge. Triplicates (or more) were performed for each sample.

#### Surface Analysis of the Delaminated Films

During mechanical tests, some of the Sandwich films delaminated. To better understand which layers delaminated and why, FTIR analysis of the delaminated surfaces was performed. A PerkinElmer spectrometer, operated in the ATR mode, was used to acquire spectra with a resolution of 2 cm^–1^ and accumulation of at least 10 scans, on minimum three different zones of the delaminated surface. The most representative spectra were used for the discussion.

#### Morphology of the Films

Cross sections of the films after the tensile test were imaged by scanning electron microscopy (SEM). A conductive 4 nm-thick coating of Au/Pd was sputtered on the samples, prior to their imaging with a Zeiss Sigma VP SEM at an operating voltage of 2 kV. At least 10 images were taken for each sample, and the most representative ones were selected.

#### Water Vapor Transmission Rate (WVTR)

Water vapor properties of the films were measured at 23 °C, 50% RH and 23 °C, 80% RH. For this purpose, 10 g of dried CaCl_2_ was placed in a 100 mL glass vial, which was closed using a cap with a hole. An aluminum mask with an exchange surface area of 4.9 cm^2^ surrounded the film that was placed on top of the glass vial to close it tightly with the holed screw cap. Vials were placed in a controlled-humidity chamber, and their mass was monitored until the increase in mass was constant. Three films were measured for each sample. The WVTR was calculated using the following equation
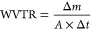
2where Δ*m* represents the increase in mass during a given time (Δ*t*) and *A* is the exposed area of the films. Experiments were conducted in triplicate. Water vapor permeability (WVP) was calculated using the thickness of the film in μm and the saturation pressure of water vapor outside the vial (*P*_sat_) depending on the relative humidity (Δ% RH).
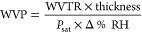
3

#### Water Surface Interaction

The water contact angle (WCA) was measured with a Theta Flex optical tensiometer (Biolin Scientific) using 5 μL water drops. Contact angles were recorded for 120 s after drop contact with the surface; three measurements were performed for each sample.

#### Grease Barrier Properties

Resistance to grease penetration was measured following a method described elsewhere.^25^ Briefly, samples (25 mm diameter) were placed between two blotting papers on top of a transparent glass plate. Then, 200 μL of “Oil Red O”-dyed olive oil was added to the upper blotting paper, and a 50 g weight was placed on top. Both the upper blotting paper and weight had a diameter of 30 mm. Grease permeation was monitored by taking images of the lower blotting paper through the glass plate with an image scanner (300 dpi, 24-bit color). The whole system was placed in a chamber at 40 °C, and periodic images were taken for 170 h.

Three parallel measurements were done for each sample.

#### Optical Properties

Transmittance of the films was measured between 200 and 800 nm with a UV–vis spectrophotometer (Shimadzu, UV-2550). Measurements were performed in triplicate.

#### Oxygen Transmission Rate (OTR)

Oxygen barrier properties were measured with a Systech Illinois M8001 oxygen permeation analyzer. Measurements were done at 23 °C and 50% RH or 80% RH, with the same humidity on both sides of the films. A 5 cm^2^ mask was used to reduce the exchange surface. The OTR was given by the device in cm^3^/m^2^·day, and the oxygen permeability (OP) was then calculated using the thickness of the film and atmospheric pressure (*P*_atm_) (eq 4). Duplicates or triplicates were measured.
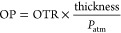
4

#### Food Test

Equal mass of commercial crackers (between 2.2 and 2.3 g) was placed in glass bottles just after opening the food packaging. The glass bottles were closed with the holed screw cap, which was covered with the different films; the exchange surface area was 4.9 cm^2^. The Sandwich and multilayer films were tested, and CNFs were used as a reference. The packaging of the crackers was used as a positive reference. The bottles were placed in a humidity chamber for 1 week at 23 °C and 80% RH. To compare the films’ functionality to protect the crackers against the high humidity in the chamber, the cracker texture was determined using a uniaxial top-load compression test after 1 week. A texture analyzer (TA XT plus, Stable Micro Systems) was equipped with a cylindrical probe with a diameter smaller than that of the crackers so that the area of compression was constant at 126.68 mm^2^. The test was performed at a constant speed of 1 mm/s, and measurement stopped when 70% strain was reached. The dry mass of the crackers was measured after the test by drying them overnight at 105 °C. Triplicates were performed.

## Results and Discussion

Herein, we prepare a multilayer film that is composed of layered biomolecules or colloids, each of them having a specific barrier property: cellulose nanofibers for the oxygen barrier, wax for the water vapor barrier, and chitin nanofibers containing lignin particles for UV shielding. The optimization of the wax layer aiming at high water vapor barrier properties was carried out in the first place followed by the combination of the different layers in one multilayer film that is thoroughly characterized. Wax particles are integrated in four different ways to a CNF film, and only the film with the best performance was used to assemble the multilayer film that also includes the chitin nanofibers and lignin particles.

### Design of the Wax Layer to Reduce Water Vapor Permeability

Five different designs including different combinations of wax and CNF mixed or layered (see Figure S3) were prepared to obtain films with low water vapor permeability. Therefore, we analyzed the water vapor transmission rate (WVTR) of the different films at two different humidities (Figure S3a). The film with the lowest WVTR was the Sandwich design with a WVTR of 5 ± 1 g/m^2^·day at 50% RH; it was composed of a continuous layer of wax surrounded by two layers of CNF. It showed that the presence of wax in the film is not enough to decrease the WVTR but that a continuous layer of wax is necessary to effectively act as a barrier for water vapor. This is in accordance with Lange and Wyser who stated that in a laminar structure, the permeability decreases exponentially, while in a particulate system, the permeability decreases linearly with the volume of additives.^26^ Compared to 50% RH, the WVTR at 80% RH increased for all the films partly due to the higher difference in water vapor pressure between outside and inside the measurement setup but also due to the swelling of the (hydrophilic) nanofiber network that favored the diffusion of water vapor through its newly expanded pores. The increase was limited for the Sandwich film, which remained at 14 ± 2 g/m^2^·day.

**Figure 2:**
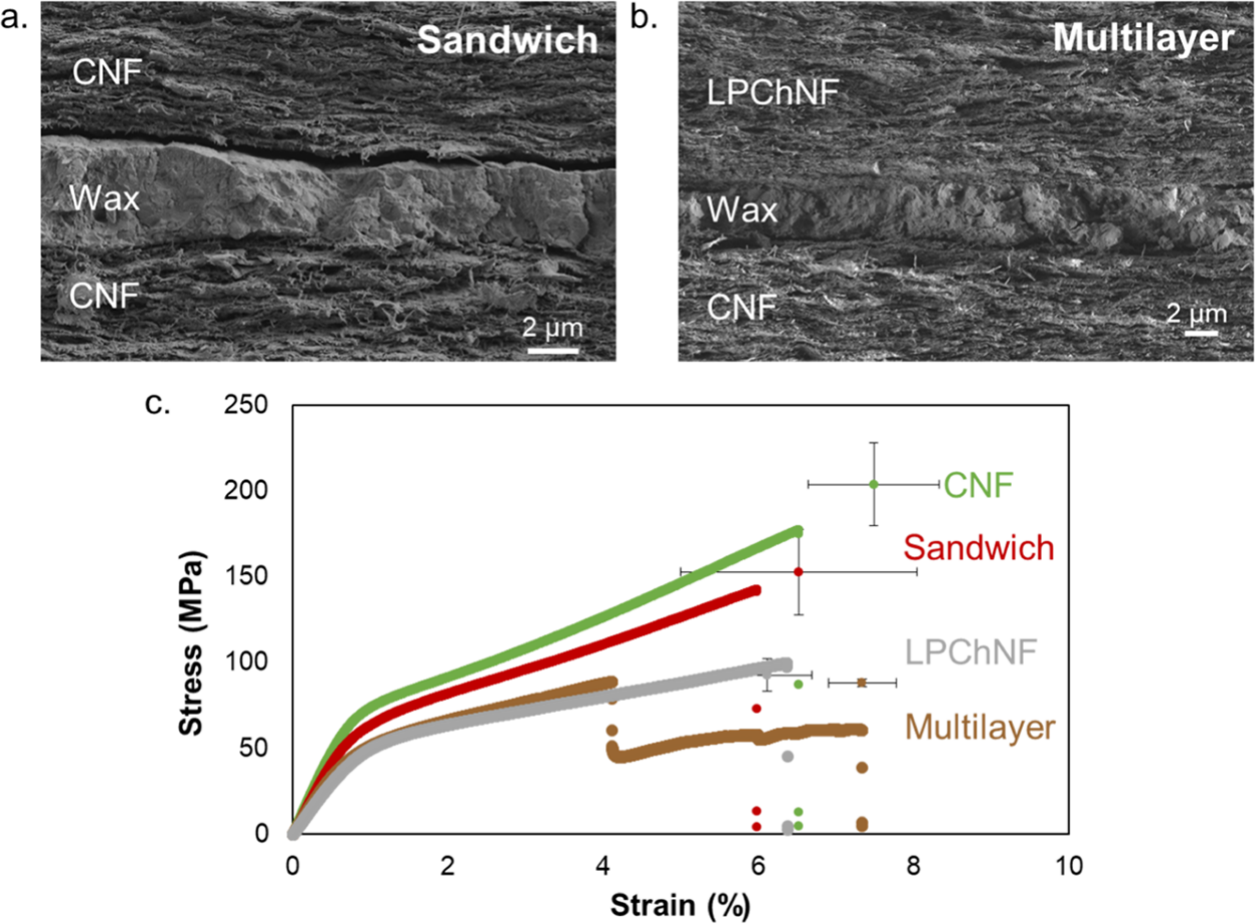
SEM image of the cross section of the sandwich (a) and multilayer (b) designs associated with the mechanical properties of the films (c). The additional point with error bars represents the average maximum strain and strength.

The presence of a continuous layer of wax in the Sandwich design was confirmed by the SEM images of the cross sections of the films (Figure 2a). The wax layer can be well-identified as a nonporous and uniform layer. The thickness of the wax layer measured from the SEM images was 3.3 ± 0.5 μm. Mechanical properties of the different films were also evaluated (Figures S4 and S5, and Supplementary Discussion 1). Films containing mixed CNFs and wax presented higher mechanical properties than the layered designs. Even though delamination took place in some Sandwich designs, their ultimate tensile strength values are comparable to those of CNFs. As demonstrated here and in the literature,^27,28^ blends with compatible components tend to have higher mechanical properties than layered materials, whereas layered materials have higher gas barrier properties. We showed that the presence of a continuous wax layer was necessary to obtain high water vapor barrier properties. Moreover, humidity had a limited impact on the WVTR, as it was driven by the wax layer, the latter being inert to humidity. In conclusion, we found the Sandwich film to be a good compromise between a higher water vapor barrier and mechanical strength; hence, it is the chosen design for the preparation of the multilayer film.

### Design of the Multilayer Film and Structural Analysis

Herein, we emphasize the versatility of layered constructs and how these designs can be used to include multiple functionalities within the same film. In this context, we decided to functionalize our Sandwich film with lignin nanoparticles. Due to the phenolic nature of lignin, the addition of lignin particles to a nanofiber film brings about UV shielding property. In a previous publication, we demonstrated that there are strong interfacial interactions between chitin nanofibers and lignin particles,^21^ which would allow high retention of the particles during filtration. On the contrary, repulsion between negatively charged lignin particles and cellulose nanofibers would lead to loss of particles during filtration and possible diffusion within the other layers. Films of chitin nanofibers containing 9% of lignin particles (LPChNF) displayed complete UV shielding and antioxidant properties at the surface of the film. To include UV light barrier properties to our high-gas barrier material, we designed a multilayer assembly by replacing one of the CNF layers in the Sandwich with LPChNF. Table S1 shows the amount of each materials used, with the wax and total nanofiber amounts being kept constant between the Sandwich and multilayer materials. The same filtration and drying methods as in the Sandwich design were used to produce the multilayer film.

Figure 2b shows the SEM image of the cross section of the multilayer film. A similar morphology as the Sandwich was observed as a dense wax layer, intercalated by two lamellar fibrous layers. Lignin particles were not visible in the top layer due to their nanometric size; however, due to the electrostatic attraction between lignin particles and chitin nanofibers (see Figure 1c),^21^ we expect the lignin particles to be present only in the ChNF layer. The wax layer thickness was 3.3 ± 0.6 μm equivalent to that of the Sandwich film. The mechanical properties of the multilayer are presented in Figure 2c. During the tensile tests, every sample of the multilayer design ruptured in two steps. We noted that the LPChNF layer was always the first one to break, which is explained by the lower strength of LPChNF at 93 ± 9 MPa compared to CNF at 204 ± 24 MPa (Figure 2c). The ultimate strength of the whole layered material was limited by its weakest part, which is a common feature among composite materials. The multilayer film during tensile testing could be compared to two single layers competing; the CNF film, having double strength and 20% higher strain than that of the LPChNF film, is expected to be more resistant. Babaei-Ghazvini et al. also obtained double fracture while measuring the tensile properties of the starch/chitosan double-layered film.^29^ While wet lamination of cellulosic materials and drying together lead to strong adhesion between layers due to the formation of strong hydrogen bonds upon drying between the layers,^30^ the hydrogen bonds between wax and CNF or ChNF were limited because of the lipidic nature of the wax. Improvement in adhesion between the layers could be achieved, for example, by adding a tie layer that has affinity toward both CNF and wax.^27^

Surface properties and interactions with liquid are important for packaging applications especially for biobased materials that are sensitive to water; hence, we measured the surface water interactions and grease penetration of the films. Figure S6 shows the water contact angle (WCA) of the different films and its evolution with time. The WCA of the top layer film was 114 ± 1°; it approximates the WCA of the wax alone, as the surface of the top layer film is mainly made of melted wax. This observation is in line with the well-known hydrophobic nature of the wax. The CNF film had the lowest WCA of 43 ± 1° followed by the Sandwich film, 55 ± 2°. The multilayer had a WCA of 70 ± 1°; it is higher than that of the Sandwich film because LPChNF was present on the surface. The WCA of LPChNF was considerably higher than the one of CNF. Both chitin and lignin have hydrophobic functional groups that decrease the hydrophilicity of the polymers; in addition, strong ionic interactions between negatively charged LP and positively charged ChNF also reduce the potential interactions with water. To further study the water interactions in the materials, wet mechanical properties should be addressed.

Grease penetration is also an essential property when packing a certain type of foods. CNFs have been reported to have barrier properties against liquid grease.^13,31^ To confirm the grease barrier properties of the films, we tested the penetration of dyed olive oil through the CNF, Sandwich, and multilayer films. After 1 week of contact at 40 °C, none of the tested films showed traces of oil penetration. This is in accordance with the literature where grease resistance properties were reported for CNF-coated paper with a coating weight as low as 11 g/m^2^ measured with the kit test method.^9^

### Multilayer Film with High Barrier Properties

**Figure 3:**
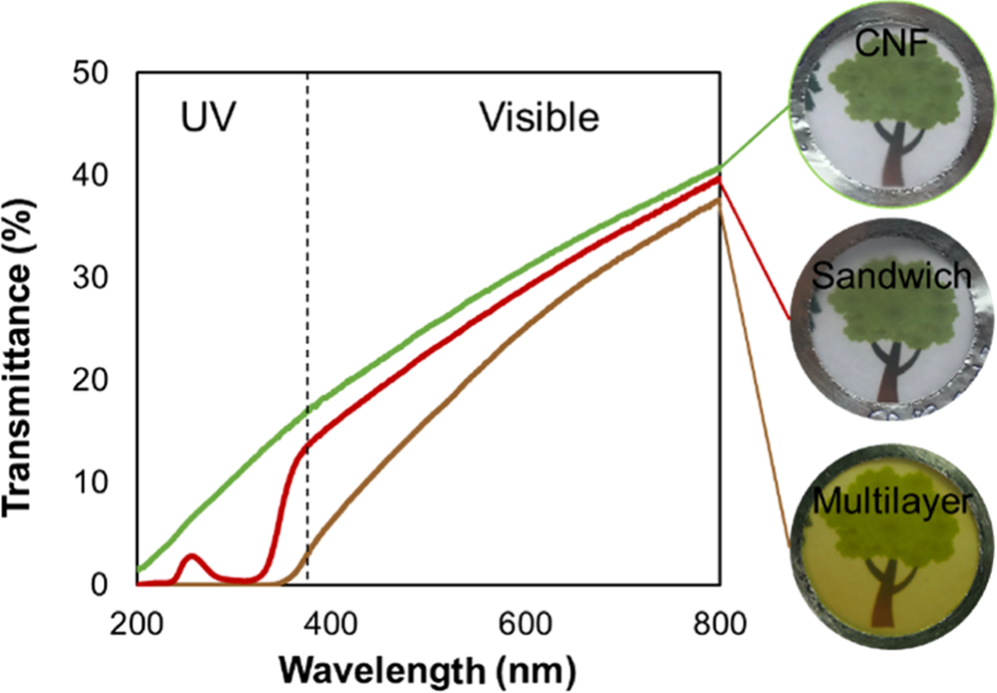
Film transmittance toward UV and visible light for the CNF, Sandwich, and multilayer films and their respective images.

The UV barrier provided by lignin in the multilayer design was assessed by UV–vis spectrophotometry (Figure 3). The films were visually homogeneous and transparent when in contact with a background. However, CNF had only a transmittance between 20 and 40% in the visible range, which shows the translucent property of the films in general. The addition of wax in the Sandwich film reduced remarkably the UV transmittance between 200–240 and 290–320 nm, but it did not induce complete UV blockage. After adding the lignin-containing layer, the transmittance in the visible range remained similar, while the transmittance in the UV range (from 200 to 350 nm) decreased to 0%. The multilayer film contained only 4% of lignin, and this was enough to provide complete UV shielding while keeping translucent properties. Similar phenomena occurred when lignin particles were added to poly(vinyl alcohol) (PVA) matrices.^32,33^ Posoknistakul et al. showed that 3% of lignin particles in PVA was needed to reach complete UV absorbance. The presence of lignin particles in the system can also improve the mechanical resistance of the film when exposed to UV light (unpublished data). In fact, UV rays are preferentially absorbed by LP, which prevents the oxidation of cellulose and the increase in its brittleness.^34^

After introducing water vapor, UV, and grease barriers, we investigated the effects of the layered combination on the oxygen transmission rate (OTR), which is an important feature in packaging, as oxygen has detrimental effects on foodstuff and can reduce their shelf life.^2^ CNFs have high barrier properties against oxygen, as they form packed layers bond together by inter- and intrafibril hydrogen bonds.^7^Table S2 presents the OTR and WVTR of our different designs and reference materials measured at 50% RH and 23 °C. It is noticed that the presence of the wax layer did not influence the OTR of the films, and it even slightly decreased it due to an overall increase in the thickness. Chitin nanofibers are also known to have high oxygen barrier properties due to the high aspect ratio of the nanofibers and their entanglement,^35^ which explains the similar OTR values between the multilayer and the Sandwich films. The WVTR of the multilayer is similar to that of the Sandwich film, as the amount of wax and the layer thickness were the same.

**Figure 4:**
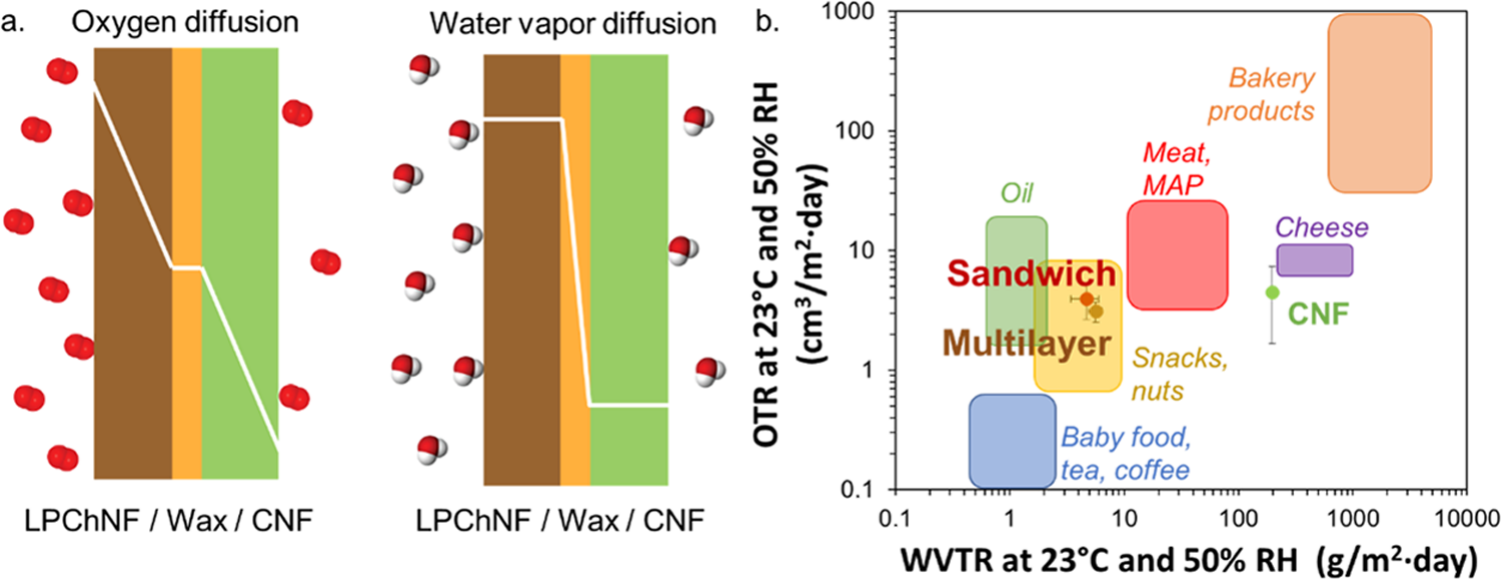
(a) Schematic of the diffusion of oxygen and water vapor throughout the multilayer film. (b) Comparison of the gas barrier properties of our films measured at 50% RH with some food requirements. Data extracted from ref (27, 37).

The permeabilities of the films were also calculated, as they do not take into consideration the thickness of the films. However, the multilayer design considers the full thickness of the film, while some layers do not contribute as a barrier, possibly resulting in permeabilities that differ from the single components. The oxygen permeability (OP) of the CNF is 2 orders of magnitude lower than the permeability of carnauba wax (Table S2); hence, the oxygen diffusion is driven by the nanofiber layers (cellulose and chitin). Their thickness, density, and crystallinity will influence the OTR of the multilayer. On the other hand, the water vapor permeability (WVP) of the CNF is 2 orders of magnitude higher than the WVP of carnauba wax (Table S2), so we expect the wax layer to be the controller of the transport of water vapor through the film. The difference in WVP between the multilayer and the wax is explained by the main contribution of the nanofibers to the thickness of the film. Figure 4a displays a schematic of the multilayer film, layers of which limit the oxygen and water vapor diffusion. Tuning the gas barrier properties is possible by changing the thicknesses of the corresponding layers, which can easily be done by increasing or decreasing the quantity of each material during the filtration process.

To evaluate the impact of humidity on the barrier properties of the films, the OTR and WVTR were also measured at 80% RH and 23 °C (Table S3). The OTR of all the films increased by 1 order of magnitude with humidity, while the WVTR increased to a smaller extent. As shown in the literature, the sensitivity to water of both cellulose and chitin nanofibers explains the increase in the OTR. At high humidity, the film swells due to the presence of water that penetrates and weakens the film cohesion by competing with the hydrogen bonds between nanofibers.^15^ Unlike cellulose and chitin, carnauba wax has a lipidic nature and does not interact with water. Still, despite its hydrophobic nature, carnauba wax contains polar groups, and its sensitivity to water is directly linked to their amount.^36^ Moreover, the increase in the OTR at high humidity was smaller for the Sandwich and multilayer materials than for the CNF reference, showing that even with its high OP, the presence of wax had an influence on the OTR at high humidity.

### Multilayer Design for Application in Food Packaging

To compare with the literature, we plotted the OTR vs WVTR graph and positioned our films on it under different humidity conditions (Figure S7) and with respect to food specificities (Figure 4b). Common polymers used for food packaging such as polyethylene (PE) and polypropylene (PP) have a low WVTR but a high OTR, so they are usually used in multilayers.^38^ It was shown that no single material can reach high barrier properties for both oxygen and water vapor, which is the main reason why multilayer assemblies have been driving the packaging market for a long time. We can see that CNF films have already sufficient oxygen barrier properties to pack several kinds of food, but their WVTR is too high for any application. Adding a wax layer and reducing the WVTR by almost 2 orders of magnitude allow us to consider biobased packaging materials for food types such as nuts and snacks regarding the gas barrier properties.^27^ Moreover, tuning the thickness of the wax layer would allow us to meet the requirements for meat and modified atmosphere packaging (MAP).

As our material is composed of several layers, it is more appropriate to compare it with similar multilayered materials. Table 1 presents the barrier properties of biobased multilayers from the literature associated with the thickness of different layers. We only show the oxygen and water vapor transmission rates, as normalizing with thicknesses to obtain the permeabilities is not appropriate for multilayered films. Most contributions added PLA as a water vapor barrier layer, as it is considered biodegradable depending on the degradation conditions and it has higher water vapor barrier property than cellulose; however, this resulted in medium water vapor barrier properties around 30–40 g/m^2^·day, as it is limited by the WVP of PLA. The coating of HDPE with CNF resulted in high barrier properties toward both oxygen and water vapor; however, at 80% RH, the OTR of the same film was 310 ± 40 cm^3^/m^2^·day due to the very low thickness of the CNF layer.^39^ Moreover, a similar film as our Sandwich design was prepared by dipping the CNF film in paraffin wax solution, which resulted in a wax/CNF/wax multilayer with a low OTR and WVTR.

**Table 1:** OTR and WVTR Measured at 50% RH for Biobased Multilayer Films in the Literature

	OTR (cm^3^/m^2^·day)	WVTR (g/m^2^·day)	thickness (μm)	references
CNF/wax/CNF	4 ± 1	5 ± 1	16/4/16	this work
CNF/wax/LPChNF	3 ± 1	6 ± 1	16/4/27	this work
paperboard/MFC/PLA	10.5	43 ± 0.4	270/8/19	Koppolu et al. 2019^10^
paperboard/CNC/PLA	6	28 ± 0.2	270/8/19	Koppolu et al. 2019^10^
PLA/(CNC/ChNF)_2_	70	65	25/4	Satam et al. 2018^35^
HDPE/CNF	0.5 ± 0.1^a^	2.2 ± 0.3^b^	48/1	Vähä-Nissi et al. 2017^39^
PLA/CNF/PLA	30^a^	50	25/30/25	Le Gars et al. 2020^37^
paper/CNF/shellac wax	4466 ± 103	6.54 ± 1.12	63/3/11	Hult et al. 2010^18^
paraffin wax/CNF/wax	0.1	40	70	Österberg et al. 2013^17^

a OTR measured at 0% RH.

b WVTR measured at 75% RH, MFC = microfibrilated cellulose, PLA = polylactic acid, CNC = cellulose nanocrystals, HDPE = high-density polyethylene.

**Figure 5:**
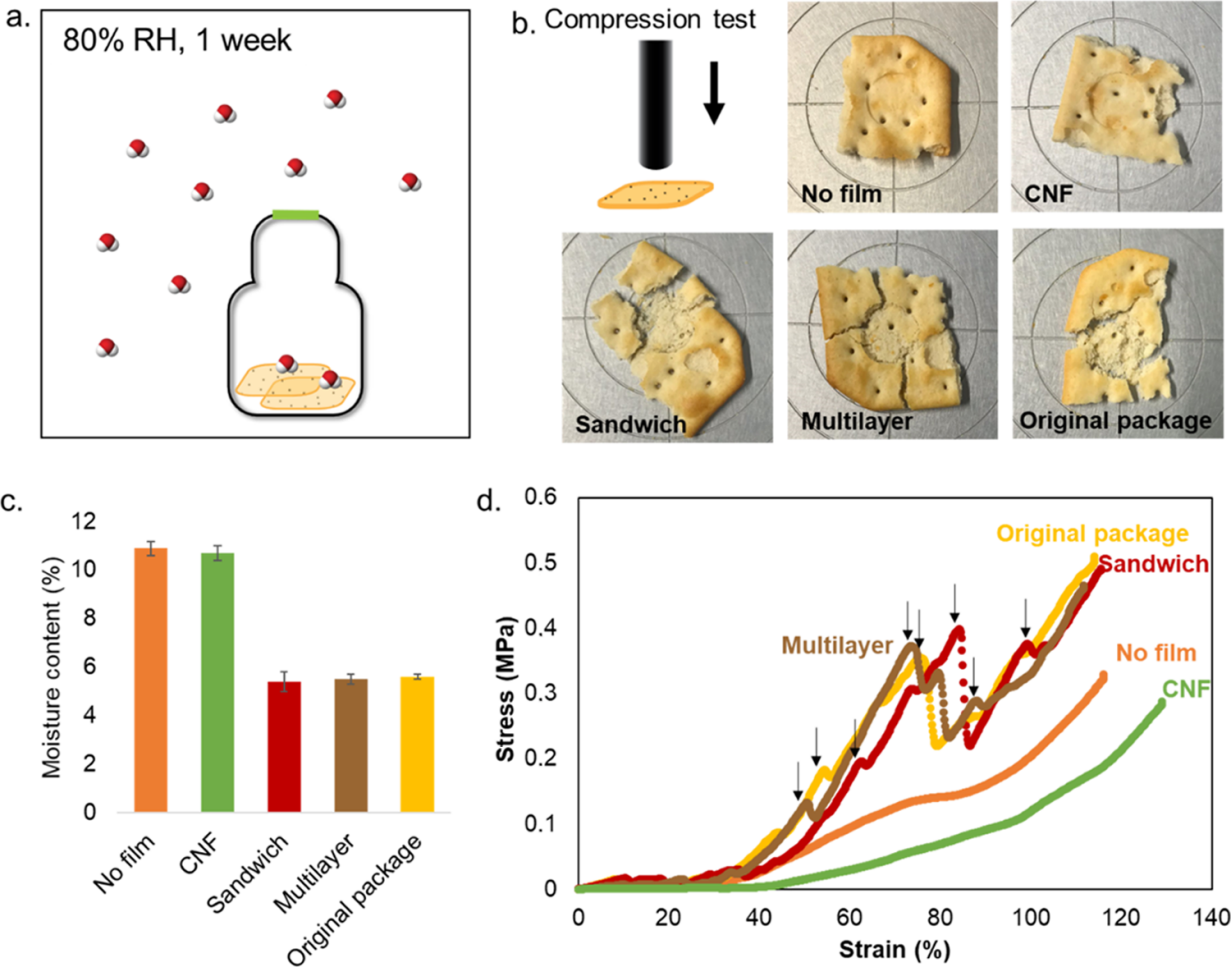
(a) Setup of the packaging test on crackers; the green line represents the tested film. (b) Images of the crackers after compression tests. (c) Moisture content of the crackers at the end of the packaging test. (d) Compression profiles of the crackers packed with the different films; the arrows point at cracks during the compression tests.

We have shown that the Sandwich and multilayer films display suitable barriers for packaging of dry food. Hence, we set up an experiment to test their effectiveness in keeping the texture of commercial crackers. The selected crackers are sensitive to humidity, becoming soft even with the slighest increase of humidity. The crackers were placed in glass bottles that are sealed with our films and the respective controls (Figure 5a). After 1 week at 80% RH, we performed compression tests on the crackers to evaluate changes in the texture (Figure 5b). The cracking texture was only observed when the crackers were protected by a high water vapor barrier film, i.e., our Sandwich and multilayer, which performed similarly to the synthetic, original package with the same experimental settings. Crackers that were unprotected or protected by CNFs showed a soft texture due to the presence of humidity. The stress and strain profile of the compression tests is displayed in Figure 5d; the cracking during the tests is indicated by arrows. In fact, cracking in the crackers corresponds to an instantaneous release of stress, which is shown by a peak in the stress–strain curve. The presence of multiple cracks during the compression test shows that the crackers were well-preserved and that their main textural property was maintained. Crackers protected by the CNF film displayed a smooth compression profile with no peak, with similar results obtained when no films were used.

The efficiency of our films is also highlighted by the final moisture content of the crackers after 1 week of being conditioned at 80% RH (Figure 5c). Crackers protected by the Sandwich and multilayer designs had a similar moisture content as crackers protected by the original package, while the one protected by the CNF film had a double moisture content.

We demonstrated that our Sandwich (CNF/wax/CNF) film can be further functionalized by including anchored nanoparticles. We exemplify with UV-blocking lignin particles, but other active molecules or nanoparticles could also be incorporated. The gas barrier properties (oxygen and water vapor) were not influenced by the presence of the functional layer. Comparison with the literature showed that as a fully biobased multilayer, our films stand out for their high barrier properties. Moreover, the packaging test on crackers was done to demonstrate the low water vapor permeability of our film and its real impact on maintaining the texture of dry food, showing a practical case to replace food packaging by biobased and biodegradable high-performance solutions.

## Conclusions

By tuning the process to incorporate carnauba wax into cellulose nanofiber films, we optimized films toward high water vapor barrier properties. A WVTR as low as 5 ± 1 g/m^2^·day was obtained when a wax layer of 3.3 μm was introduced in between two CNF layers. The high barrier properties of the CNF toward oxygen were also maintained at 4 ± 1 cm^3^/m^2^·day at 50% RH. Moreover, we demonstrated the possibility to add lignin nanoparticles to one of the nanofiber layers to provide UV shielding properties to the multilayer film while keeping translucent properties. The high gas barrier properties of the film were not impacted by the presence of functional nanoparticles. An example with dry food application demonstrated all the potential of this innovative multilayer biobased solution. Moreover, we foresee that functionalization with other kinds of active particles or molecules is possible using the same method.

Problems of adhesion between the wax layer and the cellulose or chitin layers was pointed out by delamination during the tensile testing of the films, but we point out that even with delamination, the ultimate strain of our materials was comparable to that of the CNF reference. Moreover, the addition of a tie layer to improve compatibility between the layers should be further studied. Considering the high barrier properties of the obtained films, further study on upscaling of the process is a viable next step to be done. We consider a cellulosic substrate as a support with coating or wet lamination of different suspensions (CNF, wax), the most promising idea on a short-term basis.

## Supporting Information Available

Composition, thickness, and apparent densities of the films, SEM image and DLS of the wax particles, preparation steps of the different films, SEM images of the cross sections of the CNF, Mix, Sandwich WP_CNF_, and top layer films associated with their WVTR, discussion on the mechanical properties, tensile tests of the different films, tensile tests of the Sandwich film with double fracture and FTIR of the delaminated surface of the films, water contact angles of the films’ surfaces, and OTR, OP, WVTR, and WVP of the films at 50% RH and 80% RH (PDF)
