# Supplementary material for: Multilayers of Renewable Nanostructured Materials with High Oxygen and Water Vapor Barriers for Food Packaging
Source: ACS Appl Mater Interfaces. 2022 Jun 21;14(26):30236–45. doi: 10.1021/acsami.2c07579 (PMC9815692; doi:10.1021/acsami.2c07579)
Supplement: Supplementary file 1 — am2c07579_si_001.pdf [file am2c07579_si_001.pdf]

Supporting Information

Multilayers of renewable nanostructured materials with high oxygen and water vapor barriers for food packaging

Eva Pasquier[a,b], *Bruno D. Mattos*[b], *Hanna Koivula*[c], *Alexey Khakalo*[d], *Mohamed Naceur Belgacem*[a,e], *Orlando J. Rojas*[b,f]*, *Julien Bras*[a]*

[a] Université Grenoble Alpes, CNRS, Grenoble INP (Institute of Engineering), LGP2, F-38000 Grenoble, France

[b] Department of Bioproducts and Biosystems, School of Chemical Engineering, Aalto University, P.O. Box 16300, Aalto, Espoo FIN-00076, Finland

[c] Department of Food and Nutrition and Helsinki Institute of Sustainability Science, P.O. Box 66 (Agnes Sjöobergin katu 2), University of Helsinki, FI-00014, Finland

[d] VTT Technical Research Centre of Finland Ltd., Tietotie 4E, P.O. Box 1000, FI-02044 Espoo, Finland

[e] Institut Universitaire de France (IUF), 75000 Paris, France

[f] Bioproducts Institute, Department of Chemical and Biological Engineering, Departments of Chemistry and Departments of Wood Science, University of British Columbia, 2360 East Mall, Vancouver, BC V6T 1Z3, Canada

*Corresponding authors: Julien.bras@grenoble-inp.fr, Orlando.rojas@ubc.ca

This file contains seven pages with seven figures and three tables.

Table S1. Composition, thickness, and apparent densities of the resulting films.

	CNF	Wax	ChNF	LP	Thickness	Density
	mg	mg	mg	mg	µm	
CNF	54				32 ± 2	1.35 ± 0.07
Mix	54	5.3			37 ± 3	1.26 ± 0.11
Top layer	54	5.3			47 ± 5	1.03 ± 0.15
Sandwich	54	5.3			37 ± 2	1.33 ± 0.04
Sandwich WP_{CNF}	54.5	5.3			38 ± 3	1.31 ± 0.08
Multilayer	27	5.3	27	2.7	45 ± 4	1.20 ± 0.05

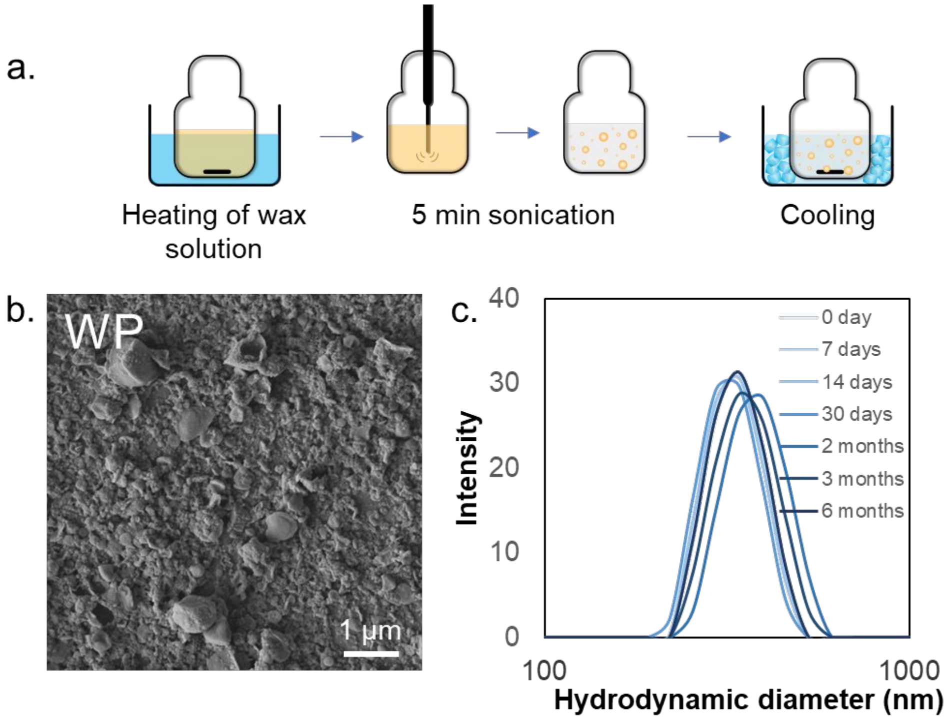

Figure S1. a. Wax particles preparation consisting of wax melting in boiling water followed by 5 min of sonication and fast cooling of the resulting emulsion. **b.** SEM image of the wax particles associated with **c.** the evolution of their size distribution over time.

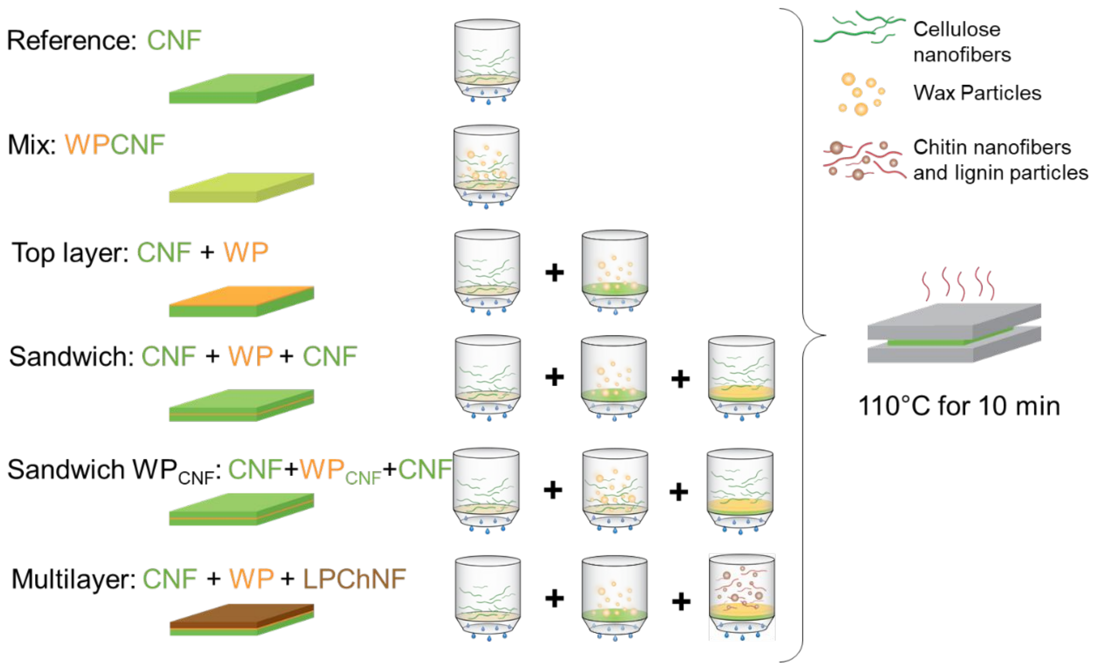

Figure S2. Different films prepared and their filtration steps. CNF, wax particles (WP) and lignin-containing chitin nanofibers (LPChNF) were assembled in several configurations aiming at coupled high oxygen and water vapor barriers. The configuration of the WP within the film was systematically assessed given its importance on promoting water vapor barrier.

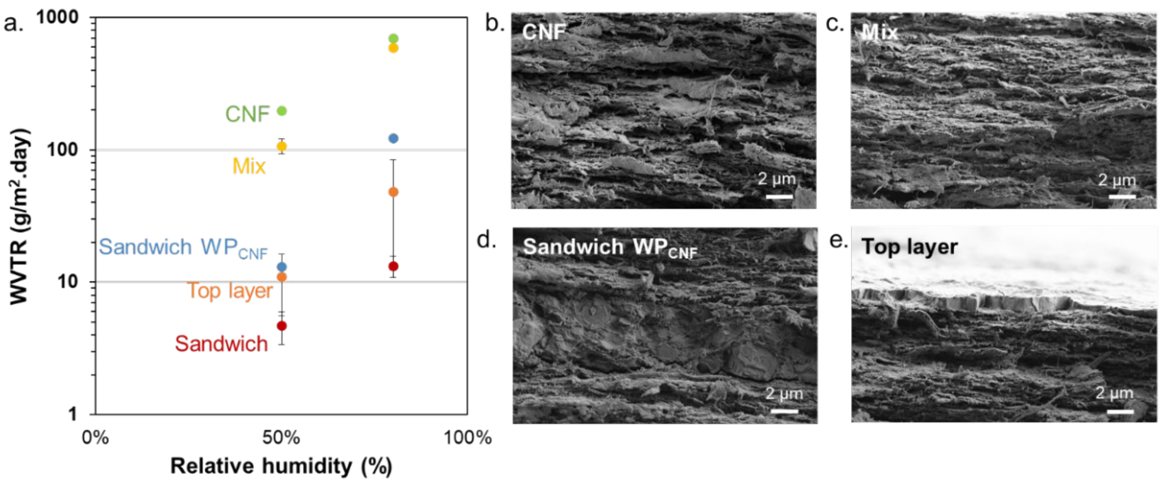

Figure S3. WVTR of the different films measured at 50% and 80% RH (**a.**) associated with the SEM images of the cross-sections of the films: **b.** CNF alone was used as reference, **c.** Mix of wax and CNF presented homogeneous and packed CNF layers comparable to the reference, **d.** Sandwich WP_{CNF} was composed of wax layer containing CNF in between two CNF layers and **e.** Top layer of wax on CNF film presented a continuous layer of pure wax.

Supplementary Discussion 1

Influence of the wax and CNF designs onto films mechanical properties.

We evaluated the mechanical properties (**Figure S3** and **S4**) of the various designs to assess the effect of the wax layer configuration on the robustness of the CNF film. **Figure S3a** shows the tensile profiles of the films. The Mix configuration had similar mechanical properties (strength, strain, and Young's Modulus) as the reference CNF film while the Sandwich and Top layer films presented similar maximum strain but lower strength and Young's Modulus. The lower modulus and strength of the Sandwich and Top layer materials could be explained by the presence of the wax layer that increases the total thickness of the film but do not contribute to the cohesion of the supporting network. In fact, when looking at the tensile profile of the *force vs displacement* (**Figure S3b**), we observed similar Young's Modulus and force at break for all the films which indicated that the mechanical properties are driven by the cellulose nanofibers layer(s), as expected.

Furthermore, during the testing of the Sandwich design, double fracture occurred in a significant number (~40%) of samples (**Figure S4a**). This behavior points at the materials breaking in two stages, first, one of the CNF layers fails followed by the second one inducing delamination of the construct. To better understand the internal fracture and to determine if the double fracture is due to a lack of cohesion of the wax layer or a lack of adhesion between the different layers, we performed FTIR analysis of the inside surface of the delaminated films. **Figure S4b** includes the FTIR spectra of both inside surfaces as well as the spectra of wax and CNF alone. The inside surface of the top part had more wax on the surface as shown by the peaks at 2916 cm^{-1} and 2847 cm^{-1} characteristic of the CH_2 stretching of the wax while the cellulose signature between 1150 cm^{-1} and 890 cm^{-1} was not visible. On the other hand, the bottom part contained significantly more cellulose than wax. Hence, the wax layer was present mostly on one side which lead to the conclusion that the adhesion between the CNF and wax layer should be further optimized. Nevertheless, even with non-interacting surfaces we reached cumulated strength values close to those of pure CNF films.

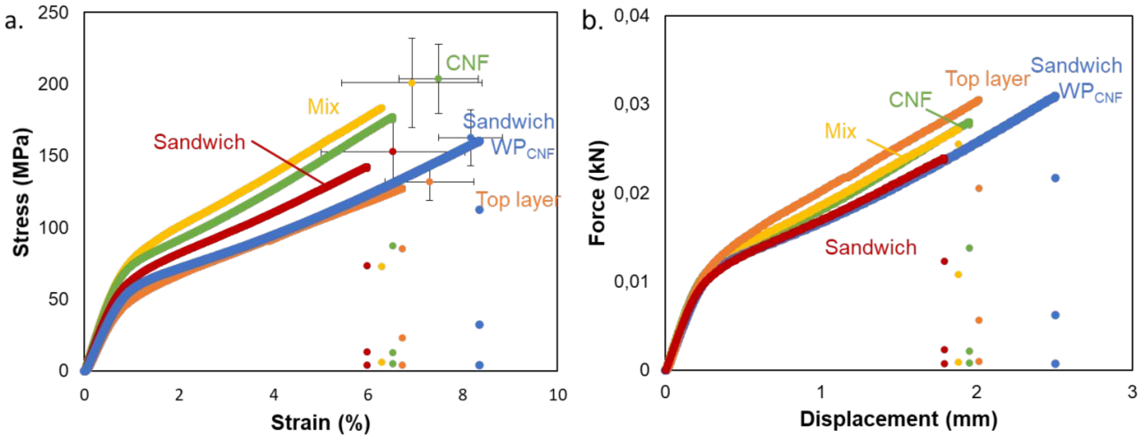

Figure S4. a. Tensile stress *vs* strain profile of the different films, as well as their average stress and strain. **b.** Force *vs* displacement of the same films during tensile tests.

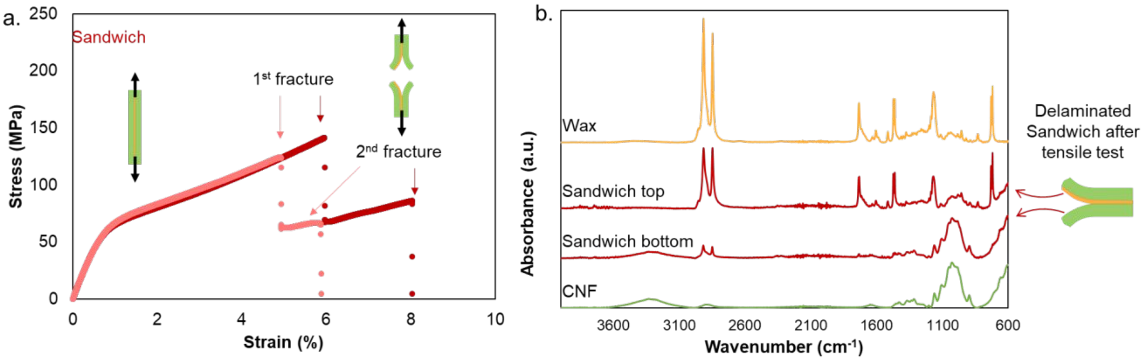

Figure S5. a. Behavior of some of the Sandwich films during tensile tests, arrows point at the different fractures. **b.** FTIR spectra of the inside surface of the films after delamination during the tensile tests associated with the spectra of wax and CNF alone.

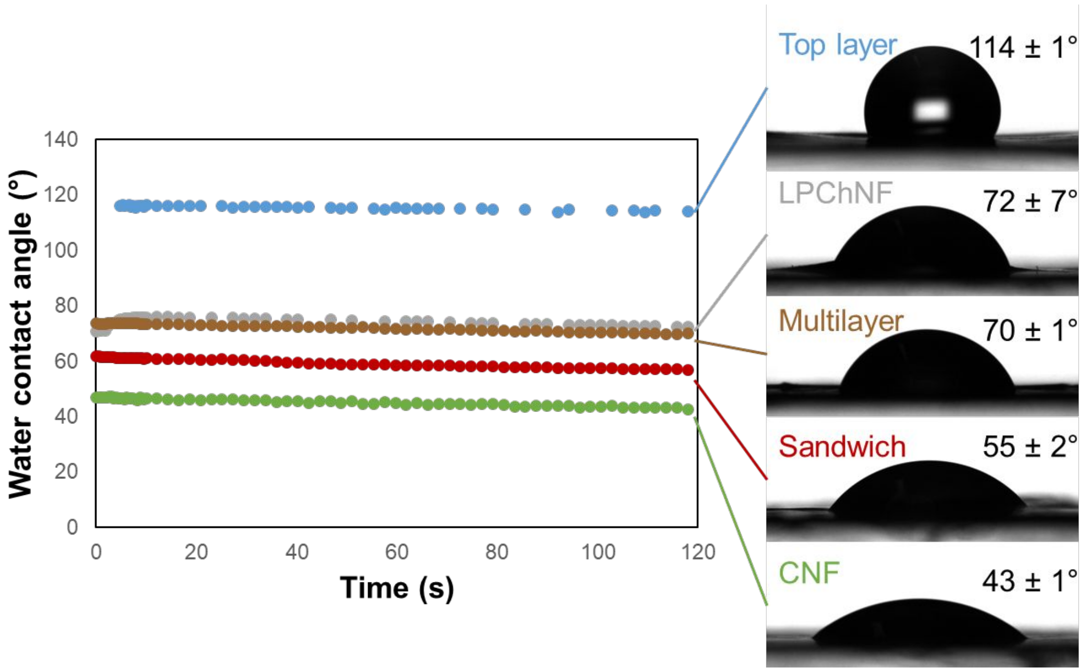

Figure S6. Water contact angle as function of time and images of the water droplets after 2 minutes of contact with the films.

Table S2. Oxygen and water vapor transmission rates measured at 50% RH and 23°C associated with their permeabilities calculated using their thicknesses. Wax permeabilities extracted from[1]

**50% RH**	OTR	OP	WVTR	WVP	Thickness
	$cm^3/m^2 \cdot day$	$\mu m \cdot cm^3/m^2 \cdot day \cdot kPa$	$g/m^2 \cdot day$	$\mu m \cdot g/m^2 \cdot day \cdot kPa$	µm
CNF	5 ± 3	1.4 ± 1.1	197 ± 3	4734 ± 511	32 ± 2
Sandwich	4 ± 1	1.5 ± 0.6	5 ± 1	121 ± 35	37 ± 2
Multilayer	3 ± 1	1.5 ± 0.4	6 ± 1	174 ± 21	45 ± 4
Wax		157		28	

Table S3. Oxygen and water vapor transmission rates measured at 80% RH and 23°C associated with their permeabilities calculated using their thicknesses.

**80% RH**	OTR	OP	WVTR	WVP	Thickness
	$cm^3/m^2·day$	$µm·cm^3/m^2·day·kPa$	$g/m^2·day$	$µm·g/m^2·day·kPa$	µm
CNF	61 ± 7	19 ± 3	695 ± 6	10501 ± 7779	32 ± 2
Sandwich	39 ± 3	15 ± 1	14 ± 2	236 ± 34	37 ± 2
Multilayer	55 ± 2	27 ± 1	16 ± 2	304 ± 39	45 ± 4

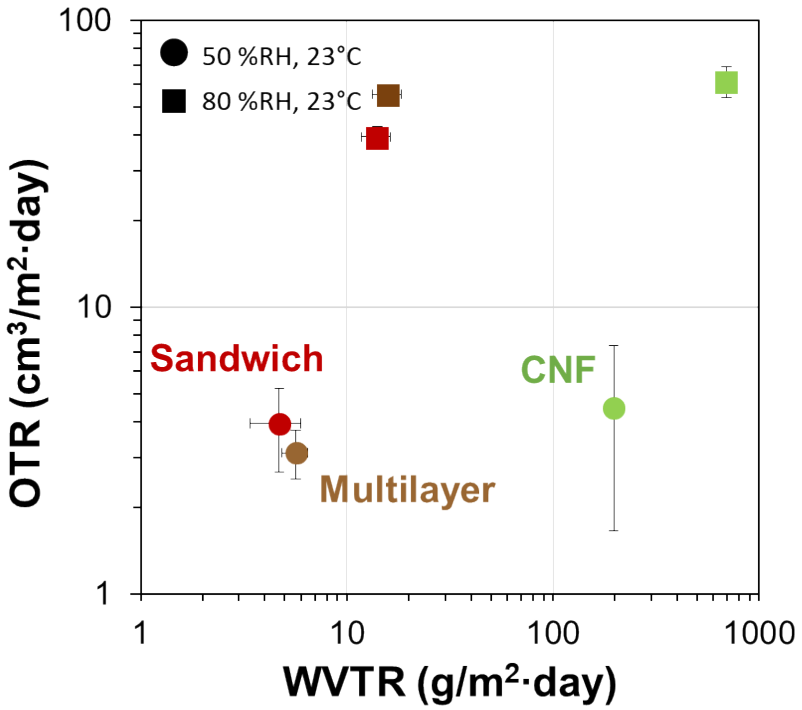

Figure S7. Barrier properties of the films at 23°C and 50% (●) and 80% (■) relative humidity.

Reference

(1) Donhowe, G.; Fennema, O. Water Vapor and Oxygen Permeability of Wax Films. *J. Am. Oil Chem. Soc.* **1993**, *70* (9), 867–873. https://doi.org/10.1007/BF02545345.
